# Positioning In Macular hole Surgery (PIMS): statistical analysis plan for a randomised controlled trial

**DOI:** 10.1186/s13063-017-2020-6

**Published:** 2017-06-13

**Authors:** Lauren Bell, Richard Hooper, Catey Bunce, Saruban Pasu, James Bainbridge

**Affiliations:** 1 Pragmatic Clinical Trial Unit, Queen Mary University of London, London, UK; 2 Department of Primary Care and Public Health Sciences, King’s College London, London, UK; 3 NIHR Biomedical Research Centre (BRC) at Moorfields Eye Hospital NHS Foundation Trust and UCL Institute of Ophthalmology, London, UK; 4 Moorfields Eye Hospital NHS Foundation Trust, London, UK; 5 Blizard Institute, Barts and The London School of Medicine and Dentistry, 4 Newark Street, London, E1 2AT UK; 6 4th Floor House, 4th Floor, Addison House, Guy’s Campus, London, SE1 1UL UK; 7 Mile End Hospital, Bancroft Road, London, E1 4DG UK

**Keywords:** Statistical analysis plan, Idiopathic macular holes, Surgery, Positioning, Recovery

## Abstract

**Background:**

The treatment of idiopathic full-thickness macular holes involves surgery to close the hole. Some surgeons advise patients to adopt a face-down position to increase the likelihood of successful macular hole closure. However, patients often find the face-down positioning arduous. There is a lack of conclusive evidence that face-down positioning improves the outcome. The ‘Positioning In Macular hole Surgery’ (PIMS) trial will assess whether advice to position face-down after surgery improves the surgical success rate for the closure of large (≥400 μm) macular holes.

**Methods/design:**

The PIMS trial is a multicentre, parallel-group, superiority clinical trial with 1:1 randomisation. Patients (*n* = 192) with macular holes (≥400 μm) will be randomised after surgery to either face-down positioning or face-forward positioning for at least 8 h (which can be either consecutive or nonconsecutive) a day, for 5 days following surgery. Inclusion criteria are: presence of an idiopathic full-thickness macular hole ≥400 μm in diameter, as measured by optical coherence tomography (OCT) scans, on either or both eyes; patients electing to have surgery for a macular hole, with or without simultaneous phacoemulsification and intraocular lens implant; ability and willingness to position face-down or in an inactive face-forward position; a history of visual loss suggesting a macular hole of 12 months’ or less duration. The primary outcome is successful macular hole closure at 3 months post surgery. The treatment effect will be reported as an odds ratio with 95% confidence interval, adjusted for size of macular hole and phakic lens status at baseline. Secondary outcome measures at 3 months are: further surgery for macular holes performed or planned (of those with unsuccessful closure); patient-reported experience of positioning; whether patients report they would still have elected to have the operation given what they know at follow-up; best-corrected visual acuity (BCVA) measured using Snellen charts at a standard distance of 6 m; patient-reported health and quality of life assessed using the National Eye Institute Visual Function Questionnaire (VFQ-25).

**Discussion:**

The PIMS trial is the first multicentre randomised control trial to investigate the value of face-down positioning following macular hole standardised surgery.

**Trial registration:**

International Standard Randomised Controlled Trials Number registry, ID: ISRCTN12410596. Registered on 11 February 2015.

United Kingdom Clinical Research Network, ID: UKCRN17966. Registered on 26 November 2014.

**Electronic supplementary material:**

The online version of this article (doi:10.1186/s13063-017-2020-6) contains supplementary material, which is available to authorized users.

## Overview

### Purpose and scope of statistical analysis plan (SAP)

The aim of this paper is to report, in detail, the planned analyses that were approved by the Trial Steering Committee for the principal research for the PIMS (Positioning In Macular hole Surgery) trial, a multicentre, interventional, comparative, randomised controlled clinical trial comparing face-down with face-forward positioning on the outcome for surgery for large macular holes. The PIMS trial is registered with ISRCTN, number 12410596 and the UKCRN portfolio, number 17966. The pilot study preceding PIMS was published in *Eye*, 2011 [1]. This pilot randomised controlled trial (RCT) explored the feasibility of a definitive trial to determine the value of face-down positioning following vitrectomy, internal limiting membrane (ILM) peeling, and C_3_F_8_ gas tamponade for full-thickness macular holes, without simultaneous phacoemulsification. The PIMS study protocol was published in *Trials*, 2015 [2]. We aim to maximise transparency of the planned analysis with the intention of eradicating misreporting or selective reporting of the trial data. We have also considered any contingences, with regards to specifying alternative analysis plans if statistical models fail to converge.

This analysis plan was written, reviewed and signed off prior to database lock, and prior to any member of the trial team having access to unmasked trial data.

### Changes from the published protocol

The protocol published in *Trials* stated that the analysis for the primary outcome would be adjusted for age and sex as fixed effects, and site as a random effect [2].

Opinion leaders questioned whether age and sex were associated with successful macular hole closure. A brief literature review highlighted a relevant study which did not find a significant association between age or sex and successful macular hole closure [3].

At the suggestion of the independent Trial Steering Committee (TSC) the PIMS management team decided to remove age and sex as covariates and to specify instead that the primary outcome would be adjusted for macular hole size (μm) and phakic lens status, with site as a random effect.

## Background and trial design

Further details of the rationale, treatment polices and design of the PIMS study are given in the study protocol.

In summary, idiopathic macular holes (macular holes) cause patients’ central vision to be blurred or distorted. Macular holes have an estimated annual incidence of 8 per 1000 individuals per year [4].

Macular holes can be treated surgically by removing the vitreous gel from the eye (vitrectomy), peeling off the ILM and then injection of a temporary gas bubble into the back of the eye. Surgery is deemed successful when the macular hole is fully closed. The current lack of evidence with which to guide patients has led to a lack of consensus among clinicians and wide variation in clinical practice. Patients are understandably confused and distressed by uncertainty and inconsistent advice.

Some surgeons advise patients to adopt a face-down position for a period immediately following surgery with the aim of improving the outcome by maintaining contact of the gas bubble with the macular hole. The duration of the face-down position varies among surgeons. However, maintaining the face-down position can be arduous for patients [5] and is associated with serious adverse events. Furthermore, evidence that face-down position treatment policy increases the closure rates of macular holes is lacking.

### Main objectives

The PIMS trial aims to determine whether the advice to position face-down improves the surgical success rate for closure of large (≥400 μm) macular holes, and thereby reduces the need for further surgery. This trial will benefit patients by providing robust evidence on the value of posturing following surgery for large macular holes, and enable health care providers and patients to make an informed choice for the best positioning to adopt after surgery.

### Trial design

The PIMS trial is a multicentre, parallel-group, superiority clinical trial with 1:1 randomisation. The trial will recruit 192 participants having surgery for large macular holes (≥400 μm), who will be randomly allocated into one of the two treatment arms:

1. Face-down positioning: subjects will be advised to maintain a face-down position for a total of at least eight consecutive or nonconsecutive hours a day for 5 days following surgery
2. Face-forward positioning: subjects will be advised to maintain a face-forward position, inactive, for at least eight consecutive or nonconsecutive hours a day for 5 days following surgery

### Participants

The PIMS trial is being conducted at nine hospitals in the UK.

Patients are deemed eligible to participate in the study if they meet the following *inclusion criteria*:

1. The presence of idiopathic full-thickness macular hole, ≥400 μm in diameter as measured by optical coherence tomography (OCT) scans, on either or both eyes
2. Patients electing to have surgery for macular hole, with or without simultaneous phacoemulsification and intraocular lens implant
3. Ability and willingness to position face-down or in an inactive face-forward position
4. Patients with a history of visual loss suggesting a macular hole of 12 months’ or less duration

Patients are deemed ineligible to participate in the study if they meet one or more of the following *exclusion criteria*:

1. Age-related macular degeneration, glaucoma, diabetic retinopathy, retinal degeneration; amblyopia; previous vitrectomy surgery (refractive error, lens opacity, and previous use of ocriplasmin are not exclusion criteria)
2. Traumatic macular hole
3. History of visual loss suggesting macular hole duration longer than 12 months
4. The presence of retinal tear identified during surgery for which postoperative positioning is advised

## Outcome measures

The primary outcome of the study is the anatomical closure of the macular hole at 3 months post surgery.

Successful closure of the macular hole will be determined by OCT scans. The scans will be anonymised and sent to two independent retinal surgeons who will independently grade the macular hole as closed; ‘open and flat’ (without a cuff of subretinal fluid); or ‘open and elevated’ (with cuff of subretinal fluid). The readers will be masked to the identity and allocated treatment of the subject. In the event of any disparity in grading, a third independent retinal surgeon, also masked to identify and treatment allocation, will arbitrate.

The categories ‘open and flat’ (without a cuff of subretinal fluid), or ‘open and elevated’ (with a cuff of subretinal fluid) will be pooled into one category of ‘open’ for the purpose of analysis.

Secondary outcomes at 3 months post surgery are:

- Further surgery for macular holes, performed or planned (yes/no)
- Best-corrected visual acuity (BCVA) measured using a Snellen chart at a standard distance of 6 m
- Patient-reported experience of positioning on a scale from 0 (very difficult) to 10 (very easy)
- Patient-reported health and quality of life assessed using the National Eye Institute Visual Function Questionnaire (VFQ-25) from 0 (worst health and quality of life) to 100 (best health and quality of life)
- Patient-reported outcome ‘Given what you now know, would you still have elected to have the operation?’ with responses Yes, No or Don’t Know

## Sample size and randomisation

### Unit of analysis

All ocular assessments relate to the study eye. In the event that a subject is having surgery for bilateral macular holes (which are not operated on simultaneously), the first eye to be operated on during the trial will be the study eye.

### Sample size

Clinical consensus is that face-down positioning would be recommended if there were a difference of 15% in success rates. This is the smallest clinically relevant treatment difference that we wish to detect. Previous research [6] indicates that successful closure of large macular holes without advice to position face-down occurs in 80% of cases. A study with 86 patients per group has 85% power and 95% confidence to detect a difference in outcome rate of 80% in the face-forward positioning arm versus 95% in the face-down positioning arm. With an anticipated 10% loss to follow-up, we are aiming to recruit 96 patients in each arm.

### Screening

The PIMS trial did not collect data on the number of participants screened for eligibility. The reason for this omission is that the proportion of patients with macular holes ≥400 μm in size is a small minority of the overall number of patients presenting with macular holes. For some hospital sites, the data collection of the number of patients screened for eligibility was considered unfeasible given their current resources.

### Randomisation and masking

Patients are randomised in a 1:1 ratio to follow either a face-forward positioning or a face-down positioning. Randomisation is stratified by site, using random permuted blocks of size 4 or 6 in equal proportions. A secure bespoke online randomisation service implemented by the Pragmatic Clinical Trials Unit performs the randomisation. Randomisation is conducted post surgery to ensure masking the surgeon to the treatment allocated. Post surgery, trial staff input the patient’s ID and details for immediate on-screen randomisation. Randomisation is provided 7 days a week, 24 h a day. Each site is provided with a unique log-in username and password to access the service. Due to the open-label nature of the treatment, postoperative clinical staff and patients are unmasked to the treatment allocation.

Investigators assessing the primary endpoint by grading of the OCT scans are masked to the treatment allocation. In the event of any disagreement between the two clinician grades, a third independent retinal surgeon, who is also masked to the treatment allocation, will arbitrate.

## Analysis methods

### General analysis principles

The primary analysis for each outcome will be by intention-to-treat, meaning that all patients on whom an outcome is available will be included in the analysis, and will be analysed according to the treatment group to which they were randomised.

Through vigilance and careful planning, the PIMS trial management team aim to achieve complete capture of all data from all patients, including patients who do not adhere to the protocol or patients who withdrawal from the trial. We acknowledge that despite our best efforts, some patients may have missing data. In accordance with the intention-to-treat principle and to avoid concerns over data-driven selection methods, we state the plan for dealing with missing data here.

Missing data is a potential source of bias, and the extent and pattern of missing data can influence the interpretation of the trial. The sample size for the PIMS trial allows for a 10% loss of follow-up, and in our results we will provide a full listing of all the reasons for patients’ withdrawal of the study. This list will be helpful in justifying the assumptions that we make in regards to missing data; however, we cannot be certain that there is a relationship between observed covariates and missing outcome data, and cannot exclude the possibility of some data missing not at random.

We will analyse the data without undertaking any sensitivity analysis if the primary outcome is missing in fewer than 5% of cases (nine patients or less). If more than nine patients’ primary outcome is missing, we will undertake sensitivity analysis to test if the missing at random assumptions are appropriate. We will use a pattern-mixture approach to model the consequences of a systematic difference between missing and nonmissing values, to see if the conclusions drawn from the PIMS study are affected when the missing-at-random assumption is violated.

All analyses will be performed using Stata (Stata Corporation, College Station, TX, USA).

### Variables measured and schedule of assessments

- The variables measured preoperatively are age, sex, ethnicity, laterality, duration of symptoms, BCVA (Best-corrected Visual Acuity), lens status (either phakic or pseudophakic), macular hole diameter on OCT scans and the Quality of Life VFQ-25 questionnaire
- The variables measured at 3 months postoperatively are BCVA, macular hole status (closed; open flat or open elevated), the Quality of Life VFQ-25 questionnaire, subject-reported experience of positioning and, if primary repair of a macular hole failed, was a second operation performed or planned?

For each comparison of outcomes according to positioning policy, the following summaries will be provided (see templates for tables in Additional file 1):

- The number of patients in each positioning policy group who are included in the analysis
- A summary measure of the outcome, by positioning policy group (mean and standard deviation (SD) for continuous outcomes, number and percentage in each category for categorical outcomes). The treatment effect (difference in means for continuous outcomes, odds ratio for binary outcomes) with its 95% confidence interval (95% CI) and *p* value

All comparisons will adjust for linear effect of macular hole size (μm) at baseline, phakic lens status at baseline, and a random effect of site (hospital). We will anonymise data reported by hospital site. Outcomes which are also assessed at baseline (BCVA and quality of life) will be analysed adjusting also for a linear effect of the baseline measurement.

Patients have a 2-week postoperative appointment, at which time clinicians may be able to identify that the macular hole closure was unsuccessful, meaning that a second operation to close the hole is required. The outcome of any participant who had a second surgery after randomisation but before the 3-month visit will be imputed as open, as it is most unlikely that the hole will close without further treatment by 3 months. These patients will not be completing secondary questionnaires.

### Participants

**Fig. 1:**
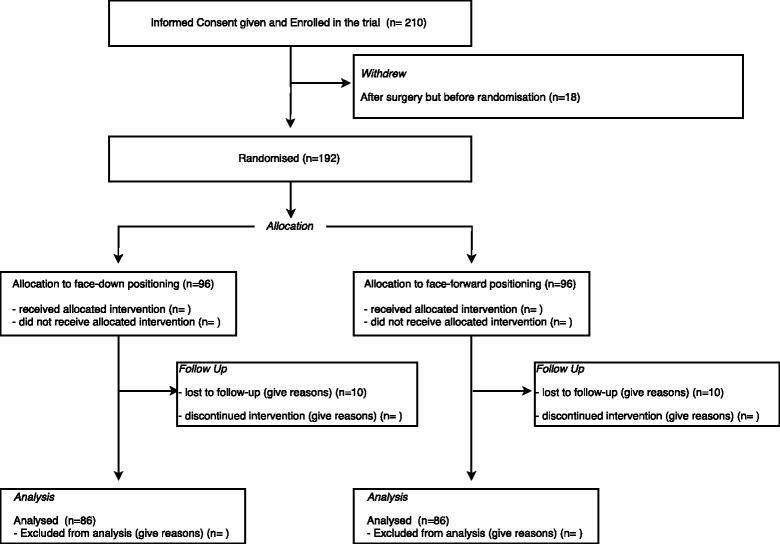
Consolidated Standards of Reporting Trials (CONSORT) flowchart for the Positioning In Macular hole Surgery (PIMS) trial, with expected numbers at each stage

We will report numbers of participants consented, randomised, and followed up in a Consolidated Standards of Reporting Trials (CONSORT) flowchart (Fig. 1). At the time of writing this SAP, recruitment of patients is on-going, and the trial teams remain unmasked; therefore, the aggregated values for the CONSORT flowchart are not provided here.

Baseline characteristics (variables measured preoperatively) will be summarised by positioning policy group. For continuous variables, we will examine the distribution of the data for symmetry, and report either the mean and SD, or the median and interquartile range (IQR) values. For categorical variables we will report the number and percentage in each category for categorical variables, with a note of numbers with missing data if any (see Additional file 1: Table S1).

### Analysis of primary outcome

This will be analysed using mixed-effects logistic regression. This analysis will be performed by the command *xtlogit* in Stata 14:

xtset site

xtlogit machole randgrp machsize i.lenstat, re

If the mixed regression model fails to converge, we will model site as a fixed effect rather than a random effect with the following command:

xtlogit machole randgrp machsize i.lenstat, fe

We will ensure that the quadrature approximation in our model is adequate and stable. First, we will run the command *quadchk* to test if our model is sensitive to changes in the number of adaptive points [7]. If the relative differences in our coefficients change by more than 1%, we will improve the quadrature approximation by increasing the number of integration points until this difference in coefficients is less than 1%. If this approach is not successful in achieving stable quadrature approximation, we will consider the command *gllamm* [8] which allows for adaptive quadrature numerical integration with the following command:

gllamm machole randgrp machsize i.lenstat, i(site) family(binom) link(logit) adapt

### Analyses of secondary outcomes

For the analysis of further surgery for macular holes, performed or planned, we will use logistic regression (*xtlogit*). Best-corrected visual acuity (BCVA), measured using Snellen charts at a standard distance of 6 m, will be transformed to a LogMAR scale with two decimal places [9], and then analysed using linear regression (*xtreg*). Measurements of BCVA corresponding to count fingers (CF), hand movements (HM), perception of light (PL), and no perception of light (NPL) will be replaced with values of 2.10, 2.40, 2.70, and 3.00, respectively. The number of patients in each BCVA category and their corresponding numerical values will be tabulated by treatment arm. For the patient-reported experience of positioning at 3 months, which is on the scale 0 (very difficult) to 10 (very easy) we will remain masked to treatment arm and consider an applicable cut-off value to dichotomise this variable and then use logistic regression (*xtlogit*). For the patient-reported outcome ‘Given what you know now, would you still have elected to have the operation?’ we will pool the responses ‘Don’t know’ and ‘No’ into one category, and use logistic regression (*xtlogit*). If the proportion of ‘Don’t know’ responses exceeds 10% (19 patients) we will undertake a sensitivity analysis by pooling the ‘Don’t know’ with ‘Yes’ responses and compare how this alternative pooling affects the odds ratio estimate. The Complications of Age-related Macular Degeneration Prevention Trial Research Group [10] observed a skewed distribution of the VFQ-25 score; therefore, we will assume that our results will be similarly skewed, and perform a logistic transformation (log(x/(100 − x))) of the VFQ-25 scores, and with this transformed outcome use linear regression (*xtreg*). When adjusting analyses of BCVA and VFQ-25 for their respective baselines we will use the same normalising transformation for the baseline measurement as for the follow-up measurement.

### Interim analyses

The PIMS Trial Steering Committee is made up of a chair, a second clinician, an independent statistician, and a lay member. There is no Data Monitoring Committee (DMC) nor any planned interim analyses for this trial due to the relatively short time span of follow-up, and minimal clinical risks. Following the guidance in the MRC’s updated terms of reference for Trial Steering Committees [11], if circumstance arise that concern the TSC, an emergency DMC, made up of independent members, will convene to review the unblinded data and advise the TSC.

### Serious adverse events

Serious adverse events (SAE), as defined in the protocol, will be tabulated and reported. The chief investigator will class the SAE as Related, which is resulting from administration of any research procedures, and/or Unexpected, that is not listed in the protocol as an expected occurrence.

#### Trial status

Recruitment began in May 2015, with nine UK hospitals participating in the trial. At the time of manuscript submission, November 2016, the PIMS trial is open to recruitment.

## Additional file

Additional file 1: Templates for PIMS results tables. (PDF 391 kb)

## Abbreviations

95% CI: 95% confidence interval
BCVA: Best-corrected visual acuity
CONSORT: Consolidated Standards of Reporting Trials
ILM: Internal limiting membrane
IQR: Interquartile range
ISRCTN: International Standard Randomised Controlled Trial Number registry
OCT: Optical coherence tomography
PIMS: Positioning In Macular hole Surgery trial
RCT: Randomised controlled trial
SAP: Statistical analysis plan
SD: Standard deviation
TSC: Trial Steering Committee
UKCRN: UK Clinical Research Network
VFQ-25: National Eye Institute Visual Function
