# Supplementary material for: Positioning In Macular hole Surgery (PIMS): statistical analysis plan for a randomised controlled trial
Source: Trials. 2017 Jun 13;18:274. doi: 10.1186/s13063-017-2020-6 (PMC5470203; doi:10.1186/s13063-017-2020-6)
Supplement: Additional file 1: — Templates for PIMS results tables. (PDF 391 kb) [file 13063_2017_2020_MOESM1_ESM.pdf]

Tables

Table 1 – Baseline characteristics

	Face-forward positioning (n=…)	***Face-down positioning (n=…)***
Age (years) – median (IQR)		
Male – no. (%)		
Ethnicity – no. (%)		
White		
Black		
Asian		
Mixed		
Other		
Laterality – Left Side – no. (%)		
Duration of symptoms (months) – median (IQR)		
BCVA (Best-Corrected Visual Acuity) – median (IQR)		
Lens Status – Phakic – no (%)		
Macular hole diameter on OCT – median (IQR)		
Quality of life VFQ-25 – median (IQR)		

Table 2 – Main results for primary outcomes

	Face-forward positioning (n=…)	***Face-down positioning (n=…)***	***Odds Ratio (95% CI)***	***P-value***
Successful closure of macular hole - n (%)				

Table 3 – Main results for dichotomous secondary outcomes

	Face-forward positioning (n=…)	***Face-down positioning (n=…)***	***Odds Ratio (95% CI)***	***P-value***
Further surgery for macular holes, performed or planned, of those with unsuccessful closure of macular hole –n(%)				
Dichotomised Patient-reported experience of positioning at 3 months- n(%)				
Patient reported outcome, if they would still have elected to have the operation				
Yes – n(%)				
Yes/Don't know (sensitivity analysis) – n(%)				

Table 4 – Main results for transformed continuous secondary outcomes

	Face-forward positioning (n=…)	***Face-down positioning (n=…)***	***Regression coefficient (95% CI)***	***P-value***
LogMAR conversion of Best corrected visual acuity (BCVA) using standard Snellen chart at 6m at 3 months - mean (sd)				
Logistic transformation of Patient-reported health and quality of life as assessed at 3 months using National Eye Institute Visual Function Questionnaire (VFQ-25) – mean (sd)				

Table 5- Main results for untransformed secondary outcomes

	Face-forward positioning (n=…)	***Face-down positioning (n=…)***
Best-corrected Visual Acuity (BCVA) using standard Snellen chart at 6m at 3 months - median (IQR)		
Patient-reported health and quality of life as assessed at 3 months using National Eye Institute Visual Function Questionnaire (VFQ-25) – median (IQR)		
Patient-reported experience of positioning at 3 months- median (IQR)		

Table 6 – Patient reported outcome, if they would still have elected to have the operation

	Face-forward positioning (n=…)	***Face-down positioning (n=…)***
Yes – n(%)		
No - n(%)		
Don't know - n(%)		
Missing - n(%)		

Table 7 – Combined surgery (removal of patient's lens as a prophylactic procedure) by site.

Site	***Combined surgery (n(%) in total)***
Site A – *n(%)*	
Site B – *n(%)*	
Site C – *n(%)*	
Site D – *n(%)*	
Site E –*n(%)*	
Site F –*n(%)*	
Site G –*n(%)*	
Site H –*n(%)*	
Site I –*n(%)*	

Table 8 – Number of participants with non-missing data

Outcome	***Face-forward positioning (n=.)***	***Face-down positioning (n=.)***
Further surgery for macular holes, performed or planned, of those with unsuccessful closure of macular hole –n(%)		
Best-corrected visual acuity (BCVA) measured using Snellen charts at a standard distance of 6 metres– n(%)		
Patient-reported experience of positioning at 3 months – n(%)		
Patient-reported health and quality of life as assessed at baseline and at 3 months using National Eye Institute Visual Function Questionnaire (VFQ-25) – n(%)		
Patient reported outcome, if they would still have elected to have the operation – n(%)		

Table 9 – Number of patients who experience complications in surgery and number of patients who with complications who withdrew from the trial, by treatment arm.

	Face-forward positioning (n=.)	***Face-down positioning (n=.)***
Number of patients with complications during surgery		
Number of patient with complications during surgery who then withdrew from the trial		

Table 10 – Number of Patients who experienced a Serious Adverse Event, by treatment arm and if this event was expected or unexpected.

	Face-forward positioning (n=.)	***Face-down positioning (n=.)***
Number of patients who experienced a Serious Adverse Event which was expected		
Number of patients who experienced a Serious Adverse Event which was not expected		

Table 11 – The number of patients for each BCVA category, and the corresponding conversion, by treatment arm.

	Face-forward positioning (n=.)	***Face-down positioning (n=.)***
Counting fingers (CF) substituted for 2.10		
Hand movement (HM) substituted for 2.40		
Perception of light (PL) substituted for 2.70		
No perception of light (NPL) substituted for 3.00		
